# Development of a self-reporting tool to obtain a Combined Index of Severity of Fibromyalgia (ICAF*)

**DOI:** 10.1186/1477-7525-8-2

**Published:** 2010-01-07

**Authors:** Miguel A Vallejo, Javier Rivera, Joaquim Esteve-Vives, Group ICAF

**Affiliations:** 1 Facultad de Psicología, Universidad Nacional de Educación a Distancia, Madrid, Spain; 2 Unidad de Reumatología, Instituto Provincial de Rehabilitación, Hospital Universitario Gregorio Marañón, Madrid, Spain; 3 Sección Reumatología, Hospital General Universitari d'Alacant, Alicante, Spain

## Abstract

**Background:**

Fibromyalgia is a syndrome with heterogeneous symptoms. The evaluation in the clinical setting usually fails to cover the complexity of the syndrome. This study aims to determine how different aspects of fibromyalgia are inter-related when measured by means of a self-reporting tool. The objective is to develop a more complete evaluation model adjusted to the complexity and multi-dimensional nature of the syndrome.

**Methods:**

Application was made of the Fibromyalgia Impact Questionnaire, the Hospital Anxiety and Depression Scale, the Brief Pain Inventory, the Fatigue Assessment Scale, the Health Assessment Questionnaire, the General Health Questionnaire (GHQ-28), the Chronic Pain Coping Inventory, the Arthritis Self-efficacy Scale and the Sleep Quality Scale. An assessment was made, on the basis of clinical interviews, case histories and specific tests, of the patient sociodemographic data, comorbidity, physical exploration and other clinical indexes. An exploratory factor analysis was made, with comparisons of the clinical index scores in extreme groups of patients.

**Results:**

The ICAF composed of 59 items was obtained, offering four factors that explain 64% of the variance, and referred to as Emotional Factor (33.7%), Physical-Activity (15%), Active Coping (9%) and Passive Coping (6.3%). A t-test between the extreme scores of these factors in the 301 patients revealed statistically significant differences in occupational status, medically unexplained syndromes, number of tender points, the six-minutes walk test, comorbidity and health care costs.

**Conclusions:**

This study offers a tool allowing more complete and rapid evaluation of patients with fibromyalgia. The test intrinsically evaluates the emotional aspects: anxiety and depression, and their impact upon social aspects. It also evaluates patient functional capacity, fatigue, sleep quality, pain, and the way in which the patient copes with the disease. This is achieved by means of a self-assessment questionnaire based on elements from well known tests.

## Background

Fibromyalgia is a syndrome with heterogeneous symptoms. The importance of each symptom has not been fully established, though generalized skeletal muscle pain, and particularly pain on digital palpation in certain points is considered by the American College of Rheumatology (ACR) to constitute a diagnostic criterion [1]. The presence of sleep disturbances and other somatic symptoms [2], together with emotional alterations [3], define a clinical condition with an important impact upon patient life [4]. In fact, fibromyalgia variably affects patient behaviour in the context of daily life activities and family, occupational and personal life, and in the way the patient copes with the limitations imposed by the disease [5].

The evaluation in the clinical setting usually fails to cover the complexity of the syndrome. Use is generally made of questionnaires such as the Fibromyalgia Impact Questionnaire (FIQ) [6] to assess the impact of the disease, the Health Assessment Questionnaire (HAQ) [7] for measuring functional capacity, questionnaires addressing fatigue such as the Fatigue Assessment Scale (FAS) [8], pain scales such as the Brief Pain Inventory (BPI) [9], and questionnaires for measuring anxiety, such as the Beck Anxiety Inventory (BAI) [10] and the State-Trait-Anxiety Inventory (STAXI) [11], or for assessing depression such as the Beck Depression Inventory (BDI) [12] or the Center for Epidemiologic Studies Depression-Scale (CES-D) [13]. Coping strategy tools are also used, such as the Multidimensional Pain Inventory (MPI) [14] or the Chronic Pain Coping Inventory (CPCI) [15], as well as evaluations of self-efficacy expectations in the form of the Arthritis Self-Efficacy Scale (ASES) [16]. There are no specific data to indicate which tools are most appropriate or which areas should be explored. The evaluated domains are varied [17], and in some cases redundant. In sum, in daily practice no tool is able to offer a good evaluation of the degree of patient impairment or of treatment efficacy. Likewise, to date no evaluative battery involving a multivariate study has been developed to address this task.

This study aims to determine how different aspects of fibromyalgia are inter-related when measured by means of a self-reporting tool. The objective is to develop a more complete evaluation model adjusted to the complexity and multi-dimensional nature of the syndrome. In addition, the study aims to evaluate the usefulness of the tool when contrasted with other information sources external to the patient, such as physical examination or different indexes showing the patient impairment.

## Methods

The study population was primarily urban and comprised women and men above 18 years of age with a diagnosis of fibromyalgia according to ACR classification criteria [1], recruited consecutively from 15 rheumatology clinics throughout the country. Patients presenting other concomitant diseases with severely impaired physical or functional capacity, rheumatic inflammatory diseases, cardiovascular or pulmonary diseases with poor aerobic capacity, uncontrolled psychiatric diseases, patients involved in litigation process, and patients included in any other clinical trial were excluded from the study. A total of 301 patients with fibromyalgia were included, 10 men and 291 women, with a mean age of 49 years.

In the first phase information was obtained throught a face-to-face interview. In the second study phase in which self-perceived health and psychosocial variables were assessed, the patients were requested to complete questionnaires, and a functional physical examination was made.

The study protocol was approved by the Clinical Research Ethics Committee of Gregorio Marañón Hospital (Madrid, Spain).

### Self-assessment questionnaires used

The questionnaires were selected from among those most commonly used to evaluate symptoms of fibromyalgia, and the principal variables related to this syndrome. We also considered the use of length of instruments to reduce the effort of the patients. The following questionnaires were used: FIQ [6,18,19]; Hospital Anxiety and Depression Scale (HADs) [20,21]; BPI [9,22]; FAS [8]; HAQ [7,23]; General Health Questionnaire (GHQ-28) [24]; CPCI [15]; ASES [25]; and a Sleep Quality Scale (SQS) where 0 = very good and 10 = very poor.

Although there are many questionnaires for evaluating depression and anxiety, the HADS is considered useful for evaluating fibromyalgia. Due to overlapping medical and psychological symptoms of the illness, this questionnaire is more suitable, since it concentrates on evaluating the cognitive aspects of anxiety and depression [17], and is a good screening test which is sensitive to change [26].

### Hetero-assessment and objective indexes

Information was obtained on the main sociodemographic variables, frequent clinical manifestations, their intensity (scored by a Likert scale 1-4), the number of medically unexplained syndromes (MUSs) and other comorbidities, and the utilization of health care and non-health care resources, laboral status and a stimated cost of the fibromyalgia impact during the year before. An evaluation was also made of patient physical condition based on the six-minutes walk test (6-MWT) [27]; the Lumbar Spine Flexion Test (LSFT) [28]; and the Patient Global Passive Mobility Assessment (PGMA) [19].

### Statistical analysis

An individual analysis was made of each of the questionnaires, taking into account their original correcting rules, along with a reliability study of the corresponding scales and, where applicable, an exploration of the scales obtained in our sample, based on exploratory factor analysis. The aim was to simplify and reduce the number of items of each scale according to their internal consistency and complementation with the global tools used. With the resulting scales and items an exploratory factor analysis was made to determine grouping of the information obtained from patients self-reports. Finally, an evaluation was made of the usefulness of the scores obtained with the new tool, in relation to other variables obtained by hetero-assessment. Calculation was made of the score obtained by each patient in relation to each of the factors obtained. To this effect, the factorial score calculation procedure was used based on the regression method. Considering the scores obtained, we explored their usefulness in differentiating the patients according to external criteria, based on t-tests between extreme groups of patients (first quartile versus the fourth quartile), along with the usefulness of the ICAF. The SPSS statistical package was used throughout.

## Results

### Questionnaires analysis

#### FIQ

An exploratory factor analysis with a Kaiser-Meyer-Olkin (KMO) index of 0.83, yielding two factors that explained 57% of the variance. The first explains 44% of the variance and groups the items 1, 3, 4, 6-8, relating to patient activities and energy. Items 1, 3 and 8 were excluded due to low correlation with the scale. The second factor (12%) was excluded, since it evaluates anxiety, depression and pain intensity, which are evaluated by other questionnaires.

#### HADs

The anxiety scale was reduced by four items, those with a lesser correlation with the scale (items 6, 8, 10 and 14), thereby leaving items 2, 4 and12. The same was applied to the depression scale, excluding items 3, 7, 9 and 11, and leaving items 1, 5, and 13.

#### BPI

The pain measurement scale was considered, discarding the item conforming the scale relating to pain interference with patient daily life, since these were evaluated by other questionnaires. Items 1 and 4 were excluded due to the low correlation with the scale. Items 2 and 3 were accepted.

#### FAS

An exploratory factor analysis yielded three factors. This contrasts with the original report on the scale [10], which only cited one factor. In our study, with a KMO of 0.882, these three factors explained 69.6% of the variance. The first factor (48.4%) is referred to as physical fatigue, with the involvement of items 1-3, 5 and 6. This was the only factor considered in our study.

#### HAQ

We considered the 8 items referred as FHAQ [29] (items 1, 3, 10, 13, 14, 18-20). Items 3, 14 and 18 showed a low correlation with the scale and were rejected. Items 1, 10, 13, 19 and 20 were accepted.

#### GHQ-28

We considered the four scales forming the questionnaire, excluding the first (somatisation scale; items 1-7). Five factors were obtained. The last two with a lesser eigenvalue (1.28 and 1.08% of the variance) corresponded to items 1 to 7, yielding two small factors of the original somatisation scale. These items were therefore excluded. In sum, we considered three scales: anxiety (8-14), social dysfunction (15-21) and depression (22-28). We excluded several items due to their low correlation with the scale, and the items accepted were: anxiety (10, 11, 13 and 14), social dysfunction (17, 18 and 21) and depression (24, 25, 27 and 28).

#### CPCI

An exploratory factor analysis yielded 14 factors. Based on the relevant analysis, the following factors and items were finally considered: coping (14, 23, 27, 49), task persistence (4, 28, 34, 51, 63), relaxation (1, 12, 59), asking for assistance and seeking social support (9, 16, 44, 57), avoidance (15, 33, 41, 42) and resting (47, 58).

#### ASES

We eliminated four items corresponding to those exhibiting a lesser correlation with the scale. Items 1, 2, 6 and 7 were thus considered.

#### SQS

The only item of this scale was included.

### Construction of the Combined Index of Severity of Fibromyalgia (ICAF)

An exploratory factor analysis was made involving the scales resulting from item reduction indicated in the section above. With a KMO of 0.86, four factors explaining 64% of the variance were obtained (table 1). The first factor was referred to as Emotional, and accounted for 33.7% of the variance. The emotional aspects relating to anxiety, depression and related social elements are involved in this factor. The second factor was referred to as Physical-Activity, and explains 15% of the variance. It covers the physical aspects of the syndrome: pain, fatigue, sleep quality and functional capacity. The third factor was referred to as Active Coping, and explains 9% of the variance. It covers positive strategies for coping with the syndrome, involving an active position on the part of the patient, and moreover includes positive expectations of self-efficacy related with the disease. Finally, the fourth factor was referred to as Passive Coping, and explains 6.3% of the variance. It addresses ways of coping with the disease centered on inactivity and on the asking for external support.

**Table 1:** Exploratory factor analysis; factor loadings by instruments used.

	Factors			
	**I** **Emotional**	**II** **Physical-Activity**	**III** **Active Coping**	**IV** **Passive Coping**

HADs_ANX_short	**.*707***	.258	-.301	.187
HADs_DEPRE_short	**.*799***	.176	-.083	.057
GHQ_ANX_SLEEP_short	**.*838***	.197	-.025	-.049
GHQ_SOCIAL_DISF_short	**.*690***	.205	-.142	.080
GHQ_DEPRE_short	**.*749***	.114	-.228	.155
SQS	.151	**.*683***	.050	-.178
FIQ_short	.243	**.*767***	.012	.298
FHAQ_short	.312	**.*565***	-.080	.411
FAS_PHYSICAL	.283	**.*603***	-.129	.394
BPI_PAIN_short	.147	**.*720***	-.025	.093
ASES_short	-.283	-.154	**.*549***	-.223
Coping	-.190	.113	**.*800***	.141
Persistence	-.045	-.125	**.*741***	-.136
Relaxation	-.082	.101	**.*758***	.270
External_Support	.004	-.062	.117	**.*664***
Avoidance	.141	.381	.017	**.*671***
Resting	.086	.124	-.058	**.*637***

Key: Factor loadings by each group of items. HADs_ANX_short: items 2,4,12 from the Hospital Anxiety and Depression Scales, HADs_DEPRE_short: 1,5,13, GHQ_ANX_SLEEP_short: 10,11,13,14 from the Goldberg Health Questionnaire-28, GHQ_SOCIAL_DISF_short: 17,18,21, GHQ_DEPRE_short: 24,25,27,28, SQS: Scale of Quality of Sleep, FIQ_short: 4,6,7 from Fibromyalgia Impact Questionnaire, FHAQ_short: 1,10,13,19,20 from the Health Assessment Questionnaire, FAS_PHYSICAL: 1,3,5,6 from the Fatigue Assessment Scale, BPI_PAIN_short: 2,3 from the Brief Pain Inventory, ASES_short: 1,2,6,7 from the Arthritis Self-Efficacy Scale, Coping: 14,23,27,49 from the Chronic Pain Coping Inventory, Persistence: 4, 28,34,51,63, Relaxation: 1,12,59, External_Support: 9,16,44,57, Avoidance: 15,33,41, 42, Resting: 47,58

We thus considered four scales that address the basic elements of the disease: the emotional aspects, which explain most of the variance (a little over 50%); the principally physical aspects, which account for a quarter of the variance; and the third and fourth scales, that address coping strategies. Two coping categories were obtained: a positive category, referred to as Active, since it contributes to improve the clinical condition through active improved self-efficacy measures; and a negative category that explains a lesser proportion of the variance and seeks to resolve the problems through passivity and by resorting to others.

The Emotional Factor comprises 17 items and yields reliability as determined by Cronbach's alpha of 0.93. Physical-Activity comprises 16 items and yields a Cronbach's alpha of 0.88. Active Coping comprises 16 items with a Cronbach's alpha of 0.85. Finally, Passive Coping comprises 10 items with a Cronbach's alpha of 0.7. A tool composed of four scales and a total of 59 items is thus formed.

Scores in the ICAF indexes are shown in Table 2. We used T scores to standardize and to facilitate the interpretation of the data, the T scores usual range is between 20 to 80.

**Table 2:** Scores of the ICAF indexes.

N = 301	Total	Emotional	Physical	Active Coping	Passive Coping
Range	47.19	44.85	49.89	49.08	47.95

Minimum	26.63	31.07	20.44	25.04	24.91

Maximum	72.82	75.92	70.33	74.13	72.86

Percentile					

25	42.53	42.04	43.52	42.51	42.98

50	50	50	50	50	50

75	56.70	56.80	56.92	57.48	56.88

Key: T scores (mean = 50, sd = 10). Total = general score including all the four factors. Emotional = score in the emotional factor, Physical: score in the physical factor, Active Coping: score in the active coping factor, Passive Coping: score in the passive coping factor

We used a total of 59 items from 9 instruments. Considering that the new instrument is in part built with this questionnaires, may be questionable to correlate this new instrument with those that were used to create it. Table 3 shows these correlations.

**Table 3:** Correlations between ICAF indexes and several instruments.

N = 301	Total	Emotional	Physical	Active Coping	Passive Coping
FIQ	.686**	.429**	.662**	-.048	.329**

FAS	.558**	.414**	.516**	-.185**	.370**

BPI	.615**	.335**	.739**	-.095	.251**

HAQ	.593**	.331**	.501**	-.093	.447**

HADs anxiety	.472**	.795**	.180**	-.096	.064

HADs depression	.463**	.722**	.254**	-.314**	.263**

CPCI	.697**	-.021	.143*	.715**	.556**

GHQ-28	.559**	.862**	.295**	-.121*	.083

ASES	-.086	-.244**	-.129*	.504**	-.304**

Key: ** p < .01, * p < .05

### Utility of the Combined Index of Severity of Fibromyalgia (ICAF)

Comparison was made of the patients not on sick leave versus those temporarily or permanently off work. Comparisons were also made of the extreme groups, quartile 1 and quartile 4, corresponding to the following variables: MUSs, number of tender points, the results of the 6-MWT, patient comorbidity, and the total cost of the disease. We also compared these comparisons with FIQ scores, to observe the differences with ICAF indexes. The results can be seen in table 4. The descriptive statistics of the sample for ICAF have been omitted, as these are standardized factor scores.

**Table 4:** Mean differences in factor scores of several patients groups.

	Mean differences in factor scores					**Mean diff**.
	**Emotional**	**Physical-Activity**	**Active Coping**	**Passive Coping**	**Total**	**FIQ**

No sick leave vs temporary leave N(170/42)	-.405*	-.236	.234	-.287	-.646*	-6,433**

No sick leave vs permanent leave N(170/35)	-.725**	-.493*	.127	-.368*	-1.546**	-10.114**

Workers. No sick leave vs temporary leave N(94/37)	-.440*	-.289	.298	-.352	-.773*	-7.491**

MUSs. 1Q vs 4Q N(81/81)	-.490*	-.161	.098	-.473*	-1.007**	-6.022**

Number of tender points. 1Q vs 4Q N(111/87)	-.172	-.496**	-.157	-.318*	-1.196**	-6.908**

Six-minutes walk test. 1Q vs 4Q N(32/73)	.348	.526*	-.229	.811**	1.346**	6.290**

Comorbidity. 1Q vs 4Q N(74/107)	-.486**	-.116	.204	-.122	-.548	-3.401

Total cost 1Q vs 4Q N(67/72)	-.603**	-.762**	.213	-.585**	-1.757**	-11.957**

Key: *p < .05, **p < .01, MUSs = medically unexplained syndromes,1Q = 1^st ^quartile, 4Q = 4^th ^quartile

## Discussion

This study offers a tool allowing more complete and rapid evaluation of fibromyalgia patients. The test intrinsically evaluates emotional aspects: anxiety and depression, and their impact upon social aspects. It also evaluates pain, fatigue, sleep quality, functional capacity and the way in which the patient copes with the disease. This is achieved by means of a self-assessment questionnaire based on elements from well known tests. The ICAF comprises four scales that offer differential information on the different aspects of the disorder. The most important scale is the so-called Emotional Factor, which generates a little over 50% of the information of the test. This stresses the role of emotional aspects (anxiety and depression) in fibromyalgia syndrome. Similar findings have been reported by other authors [30]; using the Structured Clinical Interview for DSM-IV (SCID I and II), they recorded anxiety symptoms for 32.2% of the patients, and depression in 34.8%. The patients evaluated in this study yielded a higher score for this factor when on sick leave, with increased comorbidity, a larger number of MUSs, and when the health care expenditure was higher. This Emotional Factor allows us to discriminate the patients in which fibromyalgia is more severe due to a greater social and occupational impact, and a greater variety in the associated physiological symptoms. This negative impact of emotional factors upon worsening of the disorder has been observed by other investigators in relation to different aspects ranging from coping with the disease [5] to its neuropsychological effects [31].

The second scale of the ICAF is the so-called Physical-Activity scale, which evaluates pain, fatigue, sleep quality and functional capacity, included in the main clinical domains of fibromyalgia suggested by other authors [32]. It is quantitatively less important than the Emotional Factor, but is also clearly differentiated from the latter. In relation to the external measures obtained, this scale has been shown to be sensitive to the number of tender points, the results of the 6-MWT, and the sick leave in occupationally active patients. It is also sensitive to resource expenditure. Specificity therefore exists in two physical aspects: the number of tender points and the distance covered in the 6-MWT. Our findings are in agreement with those of other authors who underscore the usefulness of the number of tender points and their clinical relevance [33-35], as well as with those who relate them to pain intensity and disability [35].

The third and fourth ICAF scales are quantitatively of little importance, though they are nevertheless of special clinical interest. The Active Coping scale is a protective factor, since it includes positive coping strategies, together with increased self-efficacy. Although no statistically significant differences were found among the studied variables, this trend as a positive factor was confirmed. Lastly, the Passive Coping scale allows us to identify a group of particularly severely affected patients. In addition to underscoring the aspects relating to sick leave, the MUSs, number of tender points and poorer performance in the 6-MWT also have an impact. A recent study [36] found that the existence of activities associated to pain symptoms, and which may be regarded as coping strategies, is related to the amount of self-reported physical activity.

A particularly relevant aspect of this study is that the criteria chosen for demonstrating the usefulness of the ICAF are independent from the aspects evaluated by the questionnaires, and moreover have been obtained objectively, establishing contrasts with the patient case histories in all cases. Based on the results obtained in our study, and adopting the caution required in generalizing the findings, the ICAF can provide an orientation as to the usefulness of administering the mentioned tests, with a view to securing adequate patient assessment in these aspects.

Today the main reference to evaluate the fibromyalgia impact is the FIQ, but this questionnaire only offers a global score. This score would not to be sufficient to discriminate between some groups of fibromyalgia patients. In table 4, we can see that the FIQ and ICAF global offer similar information in number of tender points, medically unexplained syndromes, six-minutes walk test, and several sick leave comparisons. But the ICAF scales offer valuable additional information. In the case of the MUSs (e.g.), the difference is located in the emotional factor and in the passive coping factor, but not in the physical component of the ICAF. In the variable of comorbidity neither the global index FIQ nor the ICAF show a statistical significant difference, but the ICAF emotional factor discriminates in this variable.

The ICAF instrument is longer than FIQ, but it allows to access to more complete information that could be useful to discriminate some patients or the effect of diverse therapeutical modalities commonly used in this syndrome: pharmacological, psychopharmacological, physical or psychological.

The structure of the ICAF, and its items, are derived from items of well known and scientifically solid tools. This serves to ensure maximum validity and reliability of the results obtained, and constitutes a safe starting point for examining the usefulness of the tool. On the other hand, the tool includes the main evaluative domains considered to be of importance in fibromyalgia. Some domains such as sexual activity may be considered lacking, though this has not been clearly confirmed [17]. The evaluation of cognitive alterations and dysfunctions has not been included, due to their lesser importance and the lack of tools adjusted to our setting - though this aspect requires due examination in future studies. We likewise used no generic quality of life questionnaire, since it is considered that the usual components of such questionnaires have been sufficiently evaluated.

The sample of this study consists of 291 women and 10 men. This proportion reflects the ratio between women and men commonly found tine population with fibromyalgia syndrome. However, in order to control for the sex variable we also carried out an analysis with a sample including women only. The results were similar to the ones obtained with the mixed sample (data not shown).

The ICAF must be examined by future studies involving other samples, in order to confirm the factor structure obtained its test-retest reliability and sensitivity to change, with a view to more extensive evaluation of its usefulness as an index of the severity of fibromyalgia.

## Conclusions

This exploratory study offers a tool allowing more complete and rapid evaluation of patients with fibromyalgia. The test intrinsically evaluates the emotional aspects: anxiety and depression, and their impact upon social aspects. It also evaluates patient functional capacity, fatigue, sleep quality, pain, and the way in which the patient copes with the disease. This is achieved by means of a self-assessment questionnaire based on elements from well known tests. Despite to the limitations discussed above the four factor structure obtained is an interesting tool to clarify the clinical aspects of the fibromyalgia syndrome. A test-retest reliability and validity (confirmatory factorial analysis) with other patients samples are needed to explore the clinical utility of the ICAF.

## List of abbreviations used

6-MWT: Six-minutes walk test; ACR: American College of Rheumatology; ASES: Arthritis Self-Efficacy Scale; BAI: Beck Anxiety Inventory; BDI: Beck Depression Inventory; BPI: Brief Pain Inventory; CES-D: Center for Epidemiologic Studies Depression-Scale; CPCI: Chronic Pain Coping Inventory; FAS: Fatigue Assessment Scale; FHAQ: Fibromyalgia Health Assessment Questionnaire; FIQ: Fibromyalgia Impact Questionnaire; GHQ-28: General Health Questionnaire 28; HADs: Hospital Anxiety and Depression Scale; HAQ: Health Assessment Questionnaire; ICAF: acronym in Spanish (Indice Combinado de Afectación de la Fibromialgia) of the English "Combined Index of Severity of Fibromyalgia"; KMO: Kaiser-Meyer-Olkin; LSFT: Lumbar Spine Flexion Test; MPI: Coping strategy tools are also used, such as the Multidimensional Pain Inventory; MUSs: Medically unexplained syndromes; PGMA: Patient Global Passive Mobility Assessment; SQS: Sleep Quality Scale; STAXI: State-Trait-Anxiety Inventory.

## Competing interests

This research was supported by a grant of Pfizer Laboratory and Fondo de Investigaciones Sanitarias (FIS) PI 07/0202.

There are no financial or other conflicts of interest of which we are aware.

## Authors' contributions

JR and JEV conceived the study. MAV designed the study and perform the statistical analysis. These authors revised the data obtained and draft the manuscript. MAV coordinated the analysis, results and discussion. All the authors read and approved the final manuscript.

ICAF Group authors contributed only in the data acquisition.

## Note

* ICAF is an acronym in Spanish (Indice Combinado de Afectación de la fibromialgia) of the English "Combined Index of Severity of Fibromyalgia".

## References

[B1] Wolfe F Smythe HA Yunus MD The American College of Rheumatology 1990 criteria for the classification of fibromyalgia: report of the Multicenter Criteria Committee Arthritis Rheum 1990 33 160 172 10.1002/art.1780330203 2306288

[B2] Yunus MB Fibromyalgia and overlapping disorders: the unifying concept of central sensitivity syndromes Semin Arthritis Rheum 2007 36 335 356 10.1016/j.semarthrit.2006.12.009 17350675

[B3] Thieme K Turk DC Heterogeneity of psychophysiological stress responses in fibromyalgia syndrome patients Arthritis Res Ther 2005 8 r9 10.1186/ar1863 PMC1526563 16356200

[B4] Bernard A Prince A Edsall P Quality of life issues for fibromyalgia patients Arthritis Care Res 2000 13 42 50 10.1002/1529-0131(200002)13:1<42::AID-ART7>3.0.CO;2-R 11094925

[B5] Van Middendorp H Lumley MA Jacobs JWG Van Doornen LJP Bijlsma JWJ Geenen R Emotions and emotional approach and avoidance strategies in fibromyalgia J Psychosom Res 2008 64 159 167 10.1016/j.jpsychores.2007.08.009 18222129

[B6] Burckhardt CS Clark SR Bennett RM The fibromyalgia impact questionnaire: Development and validation J Rheumatol 1991 18 728 733 1865419

[B7] Fries JF The assessment of disability: from first to future principles Br J Rheumatol 1983 22 Suppl 3 48 58 6223681 10.1093/rheumatology/xxii.suppl_1.48

[B8] Michielsen HJ De Vries J Van Heck GL Psychometric qualities of a brief self-rated fatigue measure The Fatigue Assessment Scale J Psychosom Res 2003 54 345 352 10.1016/S0022-3999(02)00392-6 12670612

[B9] Daut RA Cleeland CS The prevalence and severity of pain in cancer Cancer 1982 50 1913 1918 10.1002/1097-0142(19821101)50:9<1913::AID-CNCR2820500944>3.0.CO;2-R 7116316

[B10] Beck AT Epstein N Brown G Steer RA An inventory for measuring anxiety: psychometric properties J Consult Clin Psychol 1988 56 893 897 10.1037/0022-006X.56.6.893 3204199

[B11] Spielberger CD Gosuch RL Lushene RE STAI Manual 1975 Palo Alto, CA: Consulting Psychologists Press

[B12] Beck AT Ward CH Mendelson M Mock JE Erbaugh JK An inventory for measuring depression Arch Gen Psych 1961 4 561 571 10.1001/archpsyc.1961.01710120031004 13688369

[B13] Radloff LS The CESD scale: a self report depression scale for research in the general population Appl Psychol Meas 1977 1 385 401 10.1177/014662167700100306

[B14] Kerns R Turk D Rudy T The West Haven-Yale Multidimensional Pain Inventory (WHYMPI) Pain 1985 23 345 356 10.1016/0304-3959(85)90004-1 4088697

[B15] Jensen MP Turner JA Romano JM Strom SE The Chronic Pain Coping Inventory: development and preliminary validation Pain 1995 60 203 216 10.1016/0304-3959(94)00118-X 7784106

[B16] Lorig K Chastain RL Ung E Shoor S Holman HR Development and evaluation of a scale to measure perceived self-efficacy in people with arthritis Arthritis Rheumatol 1989 32 37 44 10.1002/anr.1780320107 2912463

[B17] Mease PJ Clauw DJ Arnold LM Goldenberg DL Witter J Williams DA Simon LS Strand CV Bramson C Martin S Wright TM Littman B Wernicke JF Gendreau RM Crofford LJ Fybromialgia syndrome. Omeract7 Workshop J Reumathol 2005 32 2270 2276 16265715

[B18] Rivera J González T The Fibromyalgia Impact Questionnaire: A validated spanish version to assess the health status in women with fibromyalgia Clin Exp Rheumatol 2004 22 554 560 15485007

[B19] Esteve-Vives J Rivera J Salvat I de Gracia M Alegre C Propuesta de una versión de consenso del Fibromyalgia Impact Questionnaire (FIQ) para la población española Reumatol Clin 2007 3 21 24 10.1016/S1699-258X(07)73594-5 21794391

[B20] Zigmond AS Snaith RP The Hospital Anxiety and Depression Scale Acta Psy Scan 1983 67 361 370 10.1111/j.1600-0447.1983.tb09716.x 6880820

[B21] Herrero MJ Blanch J Peri JM De Pablo J Pintor L Bulbena A A validation study of the hospital anxiety and depression scale (HADS) in a Spanish population Gen Hosp Psychiatry 2003 25 277 283 10.1016/S0163-8343(03)00043-4 12850660

[B22] Badia X Muriel C Garcia A Nunez-Olarte JM Perulero N Galvez R Validation of the Spanish version of the Brief Pain Inventory in patients with oncological pain Med Clin (Barc) 2003 120 52 59 10.1157/13042265 12570914

[B23] Esteve-Vives J Batlle Gualda E Reig A Grupo para la Adaptación del HAQ a la población Española Spanish Versión of the Health Assessment Questionnaire: Reliability, validity and Transcultural Equivalency J Rheumatol 1993 20 2116 2222 8014941

[B24] Goldberg D Williams P Cuestionario de salud general GHQ: (General Health Questionnaire) 1996 Barcelona, Spain: Masson

[B25] Gonzalez VM Stewart A Ritter PL Lorig K Translation and validation of arthritis outcome measures into Spanish Arthritis Rheum 1995 38 1429 1446 10.1002/art.1780381010 7575693

[B26] García-Campayo J Pascual A Alda M Marzo J Magallón R Fortes S The Spanish version of the fibrofatigue scales: validation of a questionnaire for the observer's assessment of fibromyalgia and chronic fatigue syndrome Gen Hosp Psychiatry 2006 28 154 160 10.1016/j.genhosppsych.2005.12.001 16516066

[B27] King S Wessel J Bhambhani Y Maikala R Sholter D Maksymowych W Validity and reliability of the 6 minute walk in persons with fibromyalgia J Rheumatol 1999 26 2233 2237 10529146

[B28] Mannerkorpi K Burckhardt CS Bjelle A Physical performance characteristics of women with fibromyalgia Arthritis Care Res 1994 7 123 129 10.1002/art.1790070305 7727551

[B29] Wolfe F Hawley DJ Goldenberg DL Russell IJ Buskila D Neumann L The assessment of functional impairment in fibromyalgia (FM): Rasch analyses of 5 functional scales and the development of the FM Health Assessment Questionnaire J Rheumatol 2000 27 1989 1999 10955343

[B30] Thieme K Turk DC Flor H Comorbid depression and anxiety in fibromyalgia syndrome: relationship to somatic and psychosocial variables Psychosom Med 2004 66 837 844 10.1097/01.psy.0000146329.63158.40 15564347

[B31] Suhr JA Neuropsychological impairment in fibromyalgia. Relation to depression, fatigue, and pain J Psychosom Med 2003 55 321 329 10.1016/S0022-3999(02)00628-1 14507543

[B32] Mease PJ Arnold LM Crofford LJ Williams DA Russell IJ Humphrey L Abetz L Martin SA Identifying the clinical domains of fibromyalgia: contributions from clinician and patient Delphi exercises Arthritis Rheum 2008 33 160 172 10.1002/art.23826 18576290

[B33] Coster L Kendall S Gerdle B Henriksson C Henriksson KG Bengtsson A Chronic widespread musculoskeletal pain. A comparison of those who meet criteria for fibromyalgia and those who do not Eur J Pain 2008 12 600 610 10.1016/j.ejpain.2007.10.001 18024204

[B34] Granges G Littlejohn G Pressure pain threshold in pain-free subjects, in patients with chronic regional pain syndromes, and in patients with fibromyalgia syndrome Arthritis Rheum 1993 36 642 646 10.1002/art.1780360510 8489541

[B35] Lundberg G Gerdle B Tender point scores and their relations to signs of mobility, symptoms and disability in female home care personnel and the prevalence of fibromyalgia syndrome J Rheumatol 2002 29 603 613 11908579

[B36] Mannerkorpi K Rivano-Fischer M Ericsson A Nordeman L Gard G Experience of physical activity in patients with fibromyalgia and chronic widespread pain Disabil Rehabil 2008 30 213 221 10.1080/09638280701254160 17852217

